# Hepatitis A: an epidemiological survey in blood donors, France 2015 to 2017

**DOI:** 10.2807/1560-7917.ES.2018.23.21.1800237

**Published:** 2018-05-24

**Authors:** Pierre Gallian, Valérie Barlet, Lina Mouna, Sylvie Gross, Sophie Lecam, Céline Ricard, Françoise Wind, Elodie Pouchol, Cécile Fabra, Benoit Flan, Catherine Visse, Rachid Djoudi, Elisabeth Couturier, Henriette de Valk, Pierre Tiberghien, Anne-Marie Roque-Afonso

**Affiliations:** 1 Etablissement Français du Sang Provence Alpes Côte d’Azur et Corse, Marseille, France; 2 Unité des Virus Emergents (UVE: Aix-Marseille Univ – IRD 190 – Inserm 1207 – IHU Méditerranée Infection), Marseille, France; 3 Etablissement Français du Sang Auvergne Rhône Alpes, Beynost, France; 4 AP-HP, Hôpital Paul Brousse, Virologie, CNR des Virus des hépatites à transmission entérique, INSERM U1993, Villejuif, France; 5 Etablissement Français du Sang, Saint Denis-La Plaine Stade de France, France; 6 Etablissement Français du Sang Centre Pays de Loire, Nantes, France; 7 Etablissement Français du Sang Haut de France, Lille, France; 8 Etablissement Français du Sang Occitanie, Toulouse, France; 9 LFB BIOMEDICAMENTS, Courtaboeuf, France; 10 Santé Publique France, French national public health agency, Saint-Maurice, France; 11 Université de Franche-Comté, Inserm, Etablissement Français du Sang, UMR 1098, Besançon, France

**Keywords:** France, food-borne infections, sexually transmitted infections, viral infections, hepatitis A virus, men who have sex with men - MSM, outbreaks, laboratory surveillance, epidemiology, blood donors

## Abstract

Since mid-2016, hepatitis A virus (HAV) outbreaks, involving predominantly men who have sex with men (MSM), have affected countries in Europe and overseas. In France, HAV screening of blood donations in 2017 revealed a HAV-RNA prevalence ca fivefold higher than during 2015–16 (4.42/10^6^ vs 0.86/10^6^; p = 0.0005). In 2017, despite a higher male-to-female ratio (5.5 vs 0.7) and the identification of MSM-associated outbreak strains, only one of 11 infected male donors self-reported being a MSM.

Since mid-2016, outbreaks involving mainly men who have sex with men (MSM) have been reported in European countries and overseas [1-6]. The outbreaks were associated with three genotype IA hepatitis A virus (HAV) strains: VDR_521_2016, RIVM_HAV16–090 and V16–25801. In this study, we report epidemiological and clinical findings pertaining to HAV infected blood donors before and during the recent outbreak in France.

## Blood donation screening for hepatitis A virus

In France, nucleic acid test (NAT) screening for HAV is not mandatory for blood donation but is a voluntary requirement since 2000 for plasma intended for the manufacture of plasma-derived medicinal products by LFB Biomédicaments, Courtaboeuf, France. Since 2015, HAV NAT screening is carried out, on behalf of LFB Biomédicaments, by the French transfusion public service (Etablissement Français du Sang: EFS). Although the screening requirements remain unchanged, EFS tests all blood donations for therapeutic use (pools of 48 samples until February 2017 and pools of 96 subsequently) using the duplex kit Procleix HAV/B19 assay on the Tigris System automation (Grifols Diagnostic Solutions, Inc., Emeryville, California (CA)/Hologic, Inc., San Diego, CA). This system has a 95% level of detection for HAV-RNA of ca 1 IU/mL in individual testing, and thus a 95% level of detection of ca 100 IU/mL in pool testing (96 samples). Candidates for donation with signs suggestive of infectious diseases and/or fever > 38 °C are deferred until 2 weeks after the end of symptoms. Since July 2016, MSM permanent deferral has been reduced to one year, with the exception of apheresis quarantined plasma where identical deferral rules now apply to all donors [7].

## Findings in blood donations before and during the 2017 outbreak

In mainland France, the number of reported laboratory-confirmed hepatitis A cases in the general population increased from 701 and 666 in 2015 and 2016 respectively to over 2,980 cases during the first 10 months of 2017 with a maximum in July 2017 [8]. In 2015 and 2016, a period without major hepatitis A outbreaks in France, five blood donors were found positive for HAV-RNA among 5.84 million tested-donations. The frequency of positive HAV-RNA donations was 0.86/10^6^ donations with a male to female (M/F) ratio of 0.7. Of interest, the HAV-RNA screening was able to detect the inactivated vaccine strain in two blood donors in the days (D) following vaccination (D + 1 and D + 5). These two cases were not included in our survey. In contrast, a significant increase in the number of HAV-RNA-positive blood donors was observed in 2017 with 13 positive donations resulting in a frequency of 4.4/10^6^ donations, significantly higher than in the 2015–16 period (p = 0.0005). Between April and July, frequency peaked at 9.4/10^6^ donations, at a time when the number of cases in the general population was still increasing (Figure). 

**Figure:**
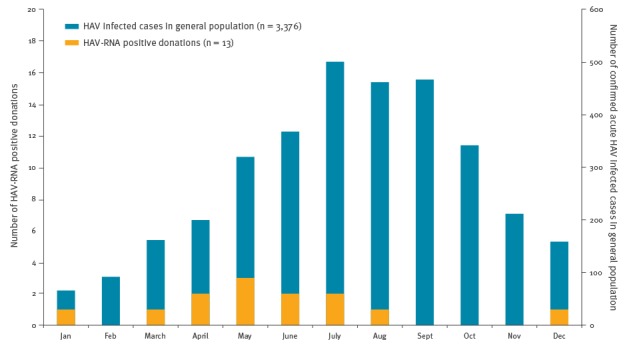
Monthly number of laboratory-confirmed hepatitis A cases stratified by cases in the general population and hepatitis A virus-RNA positive blood donations, France, 2017 (n = 3,389)

HAV: hepatitis A virus.

Data obtained through the national mandatory reporting system [8].

Blood donations found HAV-RNA-positive in 2017 involved two women and 11 men (M/F ratio = 5.5). Genotype IA outbreak-associated strains were identified in 12 of 13 cases (VDR_521_2016: n = 5; RIVM-HAV16–090: n = 7) and a genotype IIIA was identified in one case. Viral loads ranged from 1.2 to 8.59 log10 IU/mL.

None of the blood donors found HAV-RNA-positive throughout the whole study (2015–17) had symptoms at the time of donation. Sixteen donors subsequently reported clinical symptoms compatible with acute HAV infection. Among these, 11 had symptoms 2 to 12 days after donation. One donor reported symptoms at day 31 post-donation, while two had had symptoms 2 and 8 weeks before donation, respectively. Two donors failed to date the occurrence of clinical symptoms. In nine donors, systematic notification of the HAV-RNA-positive blood donation resulted in the diagnosis of asymptomatic (n = 2) or pauci-symptomatic (n = 7), previously unsuspected HAV infections.

The epidemiological investigation of the HAV-RNA-positive blood donors included questions about their job, the presence of HAV infected persons in their circle of family and friends, the presence of children < 3 years-old, travel history, and seafood consumption. Sexual risk factors were not systematically investigated. Among positive HAV-RNA blood donors, seafood consumption was the most frequently reported risk factor (n = 7). Other exposure risks were professional exposure (n = 3; including a fruit and vegetable producer, a social worker and a wastewater treatment plant worker), contact with a person infected with HAV (n = 2), travel to a hepatitis A endemic area (n = 1) and MSM (n = 1); three individuals had two risk factors. In six cases including four men in 2017, no risk factor was identified. 

**Table:** Blood donors’ characteristics, virological data and follow up of HAV infected blood products, France, 2015–2017 (n = 18 HAV-RNA-positive donations)

HAV molecular investigations					Blood products
Case number	Year of donation	Sex and age group in years	Clinical symptoms (time before or after donation)	Viral load (log IU/mL) Genotype (strain)	Transfused blood products (time after donation) *[Discarded blood products]*
1	2015	Female 49–58	Hospitalised for fever and acute abdominal syndrome (Day + 10)	4.00 IA (NN)	Red blood cell concentrate (Day + 11) *[Fresh frozen plasma]*
2	2015	Male 39–48	Asthenia during 1 week (Day - 14)	Not available IA (NN)	Pooled platelet concentrate (Day + 2) Red blood cell concentrate (Day + 9) *[Fresh frozen plasma]*
3	2016	Female 39–48	Icterus and vomiting (Day + 12)	2.48 IA (NN)	*[Red blood cell concentrate* and *fresh frozen plasma]*
4	2016	Male 39–48	Asymptomatic (NA)	2.90 IA (NN)	Pooled platelet concentrate (Day + 4) *[Red blood cell concentrate* and *fresh frozen plasma]*
5	2016	Female 39–48	Asthenia, headache, dark urine, urticaria (Day + 2)	8.40 IA (NN)	*[Fresh frozen plasma]*
6	2017	Male 18–28	Influenza-like syndrome (Day + 4) Hospitalised for acute hepatitis (Day + 10)	3.33 IIIA (NN)	*[Red blood cell concentrate* and *fresh frozen plasma]*
7	2017	Female 39–48	Febrile gastroenteritis (Day + 3) Gynaecologic pains and lumbago (Day + 8) Icterus (Day + 11)	3.94 IA (VDR_521_2016)	*[Red blood cell concentrate* and *fresh frozen plasma]*
8	2017	Male 18–28	Diarrhoea, influenza-like syndrome, mild conjunctival icterus (around Day - 60)	2.19 IA (RIVM HAV16–090)	*[Fresh frozen plasma]*
9	2017	Male 18–28	Clay-coloured stools, dark urine, digestive signs, icterus (Day + 3)	3.80 IA (VDR_521_2016)	Apheresis platelet concentrate (Day + 3)
10	2017	Male 39–48	Asthenia, mild fever (NN)	4.13 IA (VDR_521_2016)	Apheresis platelet concentrate (Day + 3) *[Fresh frozen plasma]*
11	2017	Female 29–38	Abdominal pains, asthenia, fever (Day + 7)	2.92 IA (RIVM HAV16–090)	Pooled platelet concentrate (Day + 2) *[Red blood cell concentrate* and *fresh frozen plasma]*
12^a^	2017^a^	Male 39–48	Acute hepatitis A (Day + 31)	1.2 IA (RIVM HAV16–090)	*[Fresh frozen plasma]*
13	2017	Male 59–70	Digestive signs, fever, nausea (Day + 10)	3.45 IA (RIVM HAV16–090)	Pooled platelet concentrate (Day + 2) *[Red blood cell concentrate* and *fresh frozen plasma]*
14	2017	Male 49–58	Asymptomatic (NA)	2.29 IA (VDR_521_2016)	*[Red blood cell concentrate* and *fresh frozen plasma]*
15	2017	Male 39–48	Asthenia, fever, nausea (Day + 11)	8.59 IA (VDR_521_2016)	Red blood cell concentrate (Day + 13) Pooled platelet concentrate (Day + 4) *[Fresh frozen plasma]*
16	2017	Male 18–28	Asthenia, influenza-like syndrome (NN)	5.25 IA (RIVM HAV16–090)	*[Fresh frozen plasma]*
17	2017	Male 49–58	Asthenia, dark urine, icterus, nausea (Day + 12)	4.12 IA (RIVM HAV16–090)	Pooled platelet concentrate (Day + 4) *[Red blood cell concentrate* and *fresh frozen plasma]*
18	2017	Male 29–38	Hospitalised for acute hepatitis A (Day + 8)	7.63 IA (RIVM HAV16–090)	Pooled platelet concentrate (Day + 3) *[Red blood cell concentrate* and *fresh frozen plasma]*

HAV: hepatitis A virus; NA: not applicable; NN: not known.

Viral strains were characterised as previously published [20]. Viral load in individual samples was assessed with the RealStar HAV RT-PCR Kit (Altona Diagnostics, Hamburg, Germany) with serial dilutions of the World Health Organization International Standard sample for HAV-RNA nucleic acid testing assays (NIBSC, Hertfordshire, United Kingdom) as quantification curve. Lower quantification limit was estimated at 10 IU/mL. Delay between donation date and date of identification of HAV-RNA positive donation varied but did not exceed 30 days. Upon HAV positivity detection in a pooled sample, Etablissement Français du Sang (EFS) procedure calls for a rapid identification of the HAV-infected donor.

^a^ This donor did not initially test HAV-RNA positive in pool screening, but after this person reported clinical symptoms one month post-donation, repeat testing of the individual sample found it positive. Initial negative NAT screening (pool of 96) result may be explained by a viral load (1.2 log10 IU/mL) under the 95% limit of detection (estimated for pool of 96 at around 2 log10 IU/mL).

## Follow-up of positive blood products 

Among the 12 red blood cell concentrates (RBC) from HAV-RNA-positive donations, nine were destroyed, and three already transfused before detection. All platelet concentrates (PC) (whole blood-derived pooled platelets n = 7, apheresis platelets n = 2) were transfused before availability of NAT results (2–16 days after donation). Lastly, all collected plasma were destroyed (n = 17). Haemovigilance enquiry of all 12 HAV-RNA-positive blood transfusions (3 RBC and 9 PC) revealed one case of transfusion-transmitted hepatitis A (TTHA) by the presence of HAV-RNA 7 days after transfusion in a recipient who tested RNA and IgM negative, but IgG positive with a low index ratio two days before transfusion. TTHA was confirmed by molecular comparison of involved viral strains in donor and recipient. As the survey of other recipients did not unfortunately allow full documentation of their serological and molecular status before and after transfusion, additional TTHA cases cannot be formally excluded.

## Discussion

Hepatitis A acute infection is mainly asymptomatic in children under 6 years-old, and the proportion and the severity of symptomatic forms increase with age. After a ca 30-days incubation period, the infection is typically mild and self-limited, characterised in most cases by an ‘influenza-like’ syndrome, digestive disorders and jaundice. In most cases, infection resolves within a few weeks with no reported chronic forms. However, rare cases of fulminant hepatitis with liver failure, most often fatal, are observed in elderly patients [9]. 

HAV transmission occurs through a faecal-oral route either through direct contact with an infected individual or through the consumption of contaminated water or contaminated raw food such as seafood or soiled vegetables [10]. Outbreaks occur mainly in kindergartens, in communities with precarious living conditions, or among MSM. Among the latter, outbreaks associated with sexual practices such as oro-anal or digito-anal sex have been described since the 1970s [11]. 

In this study, the involved HAV strains as well as the increased M/F ratio in 2017 suggest that sexual risk factors were under-reported and/or inadequately investigated. The recent hepatitis A virus outbreaks in Europe have involved predominantly MSM. However, during large-scale outbreaks, many individuals who do not belong to a specific risk group can get infected because HAV may spread to a larger population by direct contacts or by food handlers, especially since some infected individuals may have no symptoms. Moreover, by the end of 2017, the number of women infected by one of the outbreak-associated HAV strains had increased suggesting a spillover to non-MSM individuals [12]. In our survey, two women (cases 7 and 11) were infected by such strains.

All HAV-RNA-positive blood donors in this study were asymptomatic at the time of donation. The delay of 2 to 12 days found between donation and symptom onset in 11 of 16 HAV-RNA-positive donors who subsequently reported symptoms, is in accordance with previous TTHA published data indicating that infected blood products were collected between 6 and 18 days before the onset of clinical symptoms [13]. 

One case of TTHA was detected. TTHA is extremely rare [13-18]. This is likely due to the absence of chronic carriage, the high frequency of symptomatic forms in adults, and anti-HAV immunity in transfusion recipients as well as in blood donors. However, it should be noted that HAV seroprevalence in industrialised countries is currently decreasing. In 2008–10, HAV seroprevalence in France was 22% and 46% for the age groups of 20–29 years and 40–49 years respectively [19]. 

The risk of HAV transmission by infected blood products could be mitigated by improving donor education to immediately notify the blood bank of any symptoms occurring after blood donation as well as by blood donation HAV NAT, ideally performed shortly after donation to allow all blood products to be discarded before use. In addition to donor vaccination, these measures may also contribute to limit secondary transmission within the hospital setting or intra-familial exposure.

## Conclusions

To our knowledge, this large-scale study is the first to estimate the risk of collecting HAV-infected blood donations before and during a hepatitis A outbreak. During the 2017 HAV outbreak in France, the risk of collecting asymptomatic blood donors increased while remaining a rare event. Nevertheless, at least one TTHA occurrence was observed. Lastly, our results highlight the potential for blood donor screening to alert and/or inform of an epidemic situation.

## Erratum

In some cells of the Table, the year was incorrectly displayed. This was corrected on 25 May 2018.
